# Dynamic features of the selective pressure on the human immunodeficiency virus type 1 (HIV-1) gp120 CD4-binding site in a group of long term non progressor (LTNP) subjects

**DOI:** 10.1186/1742-4690-6-4

**Published:** 2009-01-15

**Authors:** Filippo Canducci, Maria Chiara Marinozzi, Michela Sampaolo, Stefano Berrè, Patrizia Bagnarelli, Massimo Degano, Giulia Gallotta, Benedetta Mazzi, Philippe Lemey, Roberto Burioni, Massimo Clementi

**Affiliations:** 1 Laboratorio di Microbiologia e Virologa, Università Vita-Salute San Raffaele, Milan, Italy; 2 Istituto di Microbiologia, Università Politecnica delle Marche, Ancona, Italy; 3 Unità di Biocristallografia, Istituto Scientifico San Raffaele, Milan, Italy; 4 Dipartimento di Malattie Infettive, Università Vita-Salute San Raffaele, Milan, Italy; 5 Laboratorio di Ematologia Molecolare, Istituto Scientifico San Raffaele, Milan, Italy; 6 Rega Institute, Katholieke Universiteit Leuven, Leuven, Belgium

## Abstract

The characteristics of intra-host human immunodeficiency virus type 1 (HIV-1) *env *evolution were evaluated in untreated HIV-1-infected subjects with different patterns of disease progression, including 2 normal progressor [NP], and 5 Long term non-progressor [LTNP] patients. High-resolution phylogenetic analysis of the C2-C5 *env *gene sequences of the replicating HIV-1 was performed in sequential samples collected over a 3–5 year period; overall, 301 HIV-1 genomic RNA sequences were amplified from plasma samples, cloned, sequenced and analyzed. Firstly, the evolutionary rate was calculated separately in the 3 codon positions. In all LTNPs, the 3^rd ^codon mutation rate was equal or even lower than that observed at the 1^st ^and 2^nd ^positions (p = 0.016), thus suggesting strong ongoing positive selection. A Bayesian approach and a maximum-likelihood (ML) method were used to estimate the rate of virus evolution within each subject and to detect positively selected sites respectively. A great number of N-linked glycosylation sites under positive selection were identified in both NP and LTNP subjects. Viral sequences from 4 of the 5 LTNPs showed extensive positive selective pressure on the CD4-binding site (CD4bs). In addition, localized pressure in the area of the IgG-b12 epitope, a broad neutralizing human monoclonal antibody targeting the CD4bs, was documented in one LTNP subject, using a graphic colour grade 3-dimensional visualization. Overall, the data shown here documenting high selective pressure on the HIV-1 CD4bs of a group of LTNP subjects offers important insights for planning novel strategies for the immune control of HIV-1 infection.

## Background

Virus-host relationships in human immunodeficiency type 1 virus (HIV-1) infection are characterized by a great complexity. The virus is strictly dependent on the host cell for replication, but it is constantly exposed to the immune response of the infected host. Although the innate and adaptive immune responses restrict HIV-1 replication after primary infection [1-3], efficient control of virus replication and consequent stable levels of CD4+ T-cells are observed only in a minority of patients designated long-term non progressors (LTNPs). In LTNPs virus replication is limited, suggesting that HIV-1 variants are less fit than those detectable in normal or rapid progressors in this subgroup of infected persons [4]-. Since in the absence of anti-retroviral therapy (ART), the HIV-1 replication capacity (RC) is largely related to the efficiency of viral entry [5,6]-, the selective pressure exerted either by CTL or neutralizing antibodies can account for particular evolutionary patterns in the *env *gene in LTNPs [7-10].

HIV-1 evades the immune response of the host using different mechanisms, including steric occlusion, conformational masking of critical parts of the protein, and insertions or deletions in variable loops [2,11]. Additionally, the vast majority of antibodies directed against the viral envelope recognize non-neutralizing epitopes of the glycoprotein monomers, thus probably being ineffectual against the trimeric functional complex [6,12]. Furthermore, a shifting "glycan shield" has been shown to protect the virus from neutralization by monoclonal antibodies [13-16]. Finally, many envelope surface elements are believed to serve as a decoy for the host immune system, being largely tolerant to variation with no effect on virus RC [17]. However, conserved *env *regions have been described and they are generally associated with functional properties, including virus binding to receptors and co-receptors. In particular, the CD4 binding-site (CD4bs) is believed to be a highly conserved region exposed to the solvent for ligand binding [18]-. In LTNPs, control of virus replication seems to correlate with the presence of antibodies against this critical domain, and sera from these patients show broad cross-neutralizing responses against primary HIV-1 isolates, mainly due to antibodies against this epitope [19-22].

In the past few years, a growing body of studies has investigated the HIV-1 *env *gene evolution in order to evaluate its role during the natural course of infection [19,23-27], and to identify the crucial characteristics of active and passive immunization strategies [15,18,20,28-30]-. Positively selected sites have frequently been observed within the C2-V5 region of the viral surface glycoprotein in samples from recently and chronically infected patients [1,9,10,23,24,26,27,31,32]. In the present study, a high-resolution phylogenetic analysis of partial *env *gene nucleotide sequences (C2-C5 region) was performed using samples collected over a period of 3–5 years from 7 HIV-1 infected, untreated, asymptomatic patients with different patterns of disease progression. The aim of this study was to identify conformational epitopes and sites of the viral protein surface with specific patterns of virus evolution in LTNPs.

## Results

### HIV-1 evolutionary rate in normal progressors and in long-term non progressor patients

**Figure 1:**
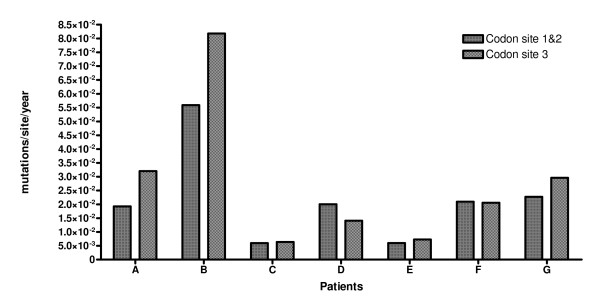
**Site specific mutation rate**. Virus mutation rate (mutations/site/year) within each patient. For each patient the mutation rate for each codon position was estimated.

Virus evolutionary rate (substitutions/site/year) within each patient was estimated separately for the first + second (μ^1st+2nd^) and third codon position (μ^3rd^) separately (Figure 1). The average viral mutation rate among all patients was estimated to be around 2.34E-02 mutations/site/year. In patients A, B (normal progressors; NP), the average mutation rate (*μ*) was significantly higher at the third position compared to that of the first and second positions (μ3^rd ^compared to μ^1st+2nd^). In all LTNPs, the third codon mutation rate was estimated to be lower or almost equal to that inferred for the other codon positions (μ3^rd ^compared to μ^1st+2nd^). This difference was found to be statistically significant when LTNP and NP results were compared with the Student t-Test (p = 0,016).

### Maximum likelihood analysis of positive selection on non recombinant data sets

We compared the fit of two sets of nested site-specific models to the data (including a neutral model that is restricted to purifying selection and an alternative model that also allows for positive selection): Model 1a vs. Model 2a and Model 7 vs. Model 8. To assess whether allowing codons to evolve under positive selection gives a significantly better fit to the data, the log likelihood values obtained for each pair of nested models were compared using the Likelihood Ratio Test (LRT) (Additional file 1). In all cases Model 2a and Model 8 were significantly favoured over Model 2a and Model 7 respectively (P < 0.001), and the empirical Bayes approach identified several positively selected sites.

**Figure 2:**
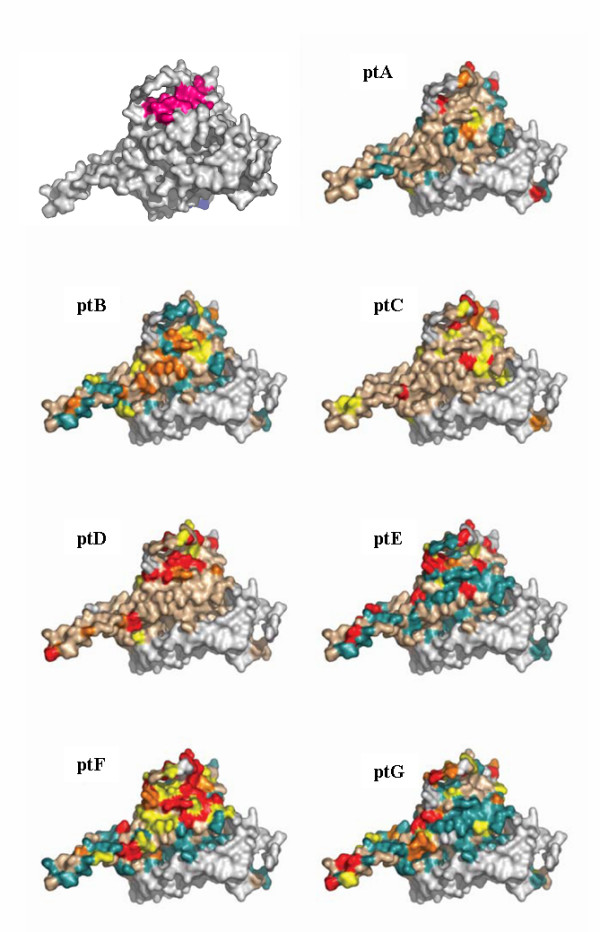
**dN/dS score visualization on the surface of gp120 (the 'silent' face of the molecule)**. Visualization of the dN/dS score (the posterior mean value derived from the Empirical Bayes approach using Model M8) onto the molecular surface of gp120 (pdb code 2B4C) using a color grade scale. Sites with no data or with a dN/dS score < 0.002 are depicted in white, sites with a dN/dS score between 0.002 and 0.15 are in light blue, sites between 0.15 and 1 are in light brown, sites with a dN/dS score between 1 and 2 are yellow, sites with a dN/dS score between 2 and 3 are orange, sites with a dN/dS score > 3 are red on the surface. A gp120 molecule was added in the upper left quadrants to localize CD4 and/or IgGb12 contact residues and the C3 alpha helix. Residues that are involved only in CD4 binding are depicted in blue, residues involved in IgGb12 binding are depicted in yellow, residues that interact both with CD4 and IgGb12 are displayed in green colour (modified from Zhou et al, 2007). The alpha helix present in the C3 region is shown in magenta.

**Figure 3:**
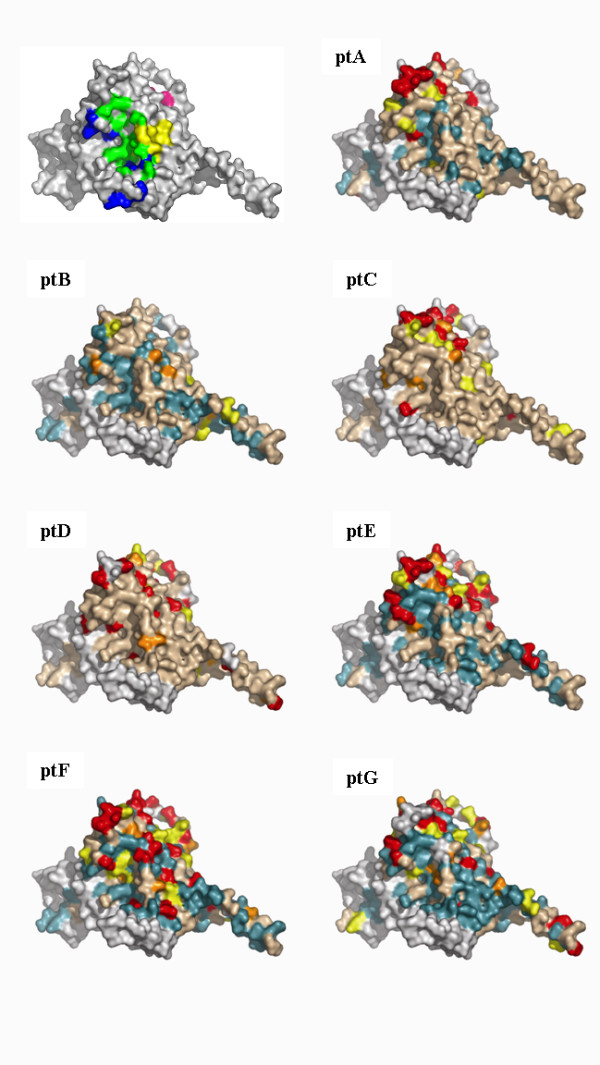
**dN/dS score visualization on the surface of gp120 (the internal portion and the CD4 binding region)**. Visualization of the dN/dS score (the posterior mean value derived from the Empirical Bayes approach using Model M8) onto the molecular surface of gp120 (pdb code 2B4C) using a color grade scale. Sites with no data or with a dN/dS score < 0.002 are depicted in white, sites with a dN/dS score between 0.002 and 0.15 are in light blue, sites between 0.15 and 1 are in light brown, sites with a dN/dS score between 1 and 2 are yellow, sites with a dN/dS score between 2 and 3 are orange, sites with a dN/dS score > 3 are red on the surface. A gp120 molecule was added in the upper left quadrants to localize CD4 and/or IgGb12 contact residues and the C3 alpha helix. Residues that are involved only in CD4 binding are depicted in blue, residues involved in IgGb12 binding are depicted in yellow, residues that interact both with CD4 and IgGb12 are displayed in green colour (modified from Zhou et al, 2007). The alpha helix present in the C3 region is shown in magenta.

Site specific dN/dS values for each patient and the entropy value for each position along the sequence were calculated (data not shown). Subsequently, a color-grade 3-dimensional visualization of the dN/dS score (the posterior mean value derived from the Empirical Bayes approach using Model M8) was generated (Figure 2 and 3). Using Model 8, the following numbers of sites with a dN/dS ratio higher than 1 were observed: patient A: 24; patient B: 33, patient C: 53; patient D: 45; patient E: 45; patient F: 81 patient G: 52. The following number of sites with dN/dS > 2 were observed: patient A: 15; patient B: 23, patient C: 27; patient D: 36; patient E: 33; patient F: 56 patient G: 34. The following numbers of sites with a dN/dS ratio higher than 3 were observed: patient A: 13; patient B: 0, patient C: 19; patient D: 25; patient E: 23; patient F: 42; patient G: 17.

The following number of sites with a posterior probability of being under positive selection > 95% and > 99%, respectively, were identified: patient A: 6 and 4; patient B: 7 and 1; patient C: 8 and 3; patient D: 10 and 7; patient E: 9 and 5; patient F: 23 and 11; patient G: 8 and 2. Selective constraints appear to act along all the proteic sequence in all patients. In all patients, positively selected sites appeared to be unevenly distributed. In particular the majority of sites were located in C3 and in V4, where many N-linked glycosylation sites are known to be present and used to protect from antibody mediated neutralization [30].

**Figure 4:**
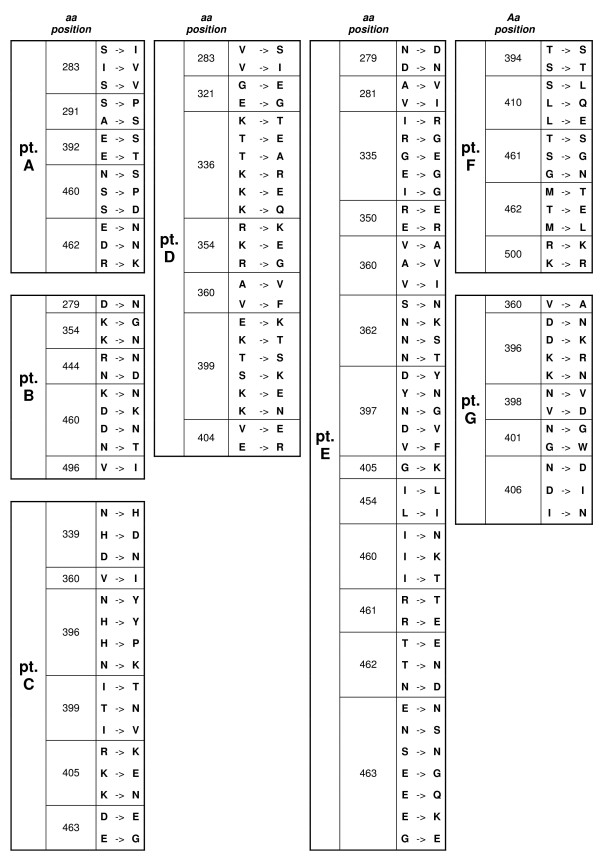
**Positively selected sites identified along internal branches**. Amino acid (aa) positions are indicated according to HXB2 sequence.

To examine the molecular footprint of deleterious mutational load on within-host evolution, and its putative impact on the identification on positively selected sites, we tested for differences in selective pressure among internal and external branches in each patient. dN/dS estimates were almost always higher on external branches compared to internal branches, but only for three patients this was statistically supported by the LRT model comparison (see Additional file 2). When the internal-external differences were tested on the data combined for all patients, however, a higher dN/dS on external branches (0.46 for internal vs 0.78 for external) was strongly supported by the LRT (< 0.001). This analysis confirms that external branches are subject to deleterious load, which might result in an elevated dN/dS ratio for these branches [33]. When we inferred the sites under selection only for the internal branches using the Fixed Effects Likelihood (FEL), several of the sites identified using the previous models were confirmed to be under positive selection (Figure 4).

For the 5 patients for which the HLA typing was obtained (see below), the majority of positively selected sites were localized outside the known HLA class I linear epitopes except for patients B, C, and E, where residues immediately next to or belonging to an HLA-A11 epitope were identified (position 339 to 350). In particular, in patient B and E residues 344Q (that is also exposed on the surface) and 346A and position 339N in patient C was inferred to be under positive selection.

### 3-dimesional analysis of the dN/dS score

**Figure 5:**
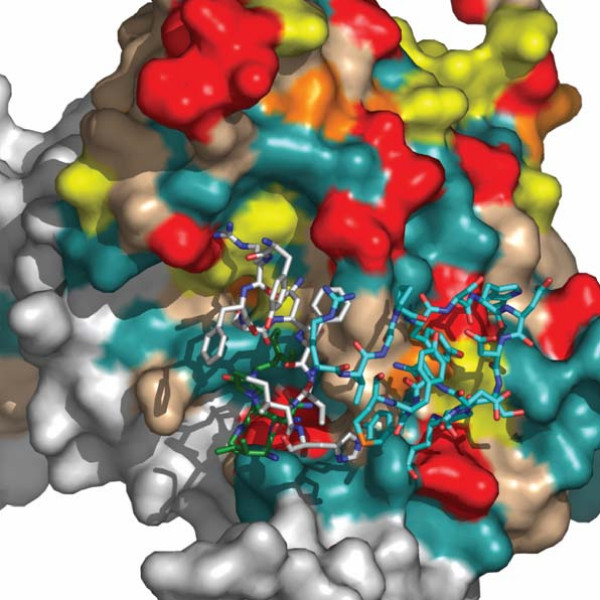
**dN/dS score visualization on the surface of gp120 (a close-up view of the interaction site between gp120 of patient F and the IgGb12 heavy chain (pdb code NY7))**. Visualization of the dN/dS score (the posterior mean value derived from the Empirical Bayes approach using Model M8) onto the molecular surface of gp120 (pdb code 2B4C) using a color grade scale. Sites with no data or with a dN/dS score < 0.002 are depicted in white, sites with a dN/dS score between 0.002 and 0.15 are in light blue, sites between 0.15 and 1 are in light brown, sites with a dN/dS score between 1 and 2 are yellow, sites with a dN/dS score between 2 and 3 are orange, sites with a dN/dS score > 3 are red on the surface. Residues that are involved only in CD4 binding are depicted in blue, residues involved in IgGb12 binding are depicted in yellow, residues that interact both with CD4 and IgGb12 are displayed in green colour (modified from Zhou et al, 2007). The alpha helix present in the C3 region is shown in magenta. The carbon atoms of CDR1, CDR2 and CDR3 are coloured white, green and cyan respectively. The amino acid residues are shown as sticks. Of note, the binding region of the broadly neutralizing antibody overlaps the positively selected sites in the patient G derived structure.

A 3-dimensional visualization of the posterior mean dN/dS value was generated using a color grade scale. Both on the CD4 binding site and on the outer domain of the molecule the majority of sites appeared as under purifying selection (Figures 2, 3 and 5, light blue areas), especially in patients C, D, and E. In many cases, amino acids that were identified as under positive selection along the gp120 linear sequence, defined clusters on the surface, suggesting their role in conformational epitopes presented on exposed antigenic areas. In all patients a high level of variation was observed in the C3 region, where an α-helix (position 335 to 350) is located and exposed on one side to the solvent and can be recognized by humoral immune defences. On the outer domain of gp120, many clusters were identified in all patients, but with a different distribution. A conformational epitope was identified in patient D, which was defined by Lys337, Ser334, Ala336, Asn339, Asn340 and Gln344. In patient F, a linear epitope in the C3 region that is exposed on the surface was identified and formed by Lys362, Glu363, Ser364 and Ser365. Another wide site of positive selection appeared to be formed by Glu269, Asn289, Ser291, Lys337, Gln340, Lys343, Gln344, and located on the outer surface. In patient G, the exposed surface harboured only two residues under positive selection: Ile371 and Gly471, which cluster together on the 3-D structure.

All patients had positively selected sites in the V3 region, specifically patient F (5 sites with a dN/dS > 1 located both on the tip and at its base). In all patients, no sites were identified among known CD4 induced epitopes.

### Analysis of the CD4 binding site

Positively selected sites were identified in the CD4 binding region in patients C, D, E and F, but not in patients A and B, where almost all positively selected sites were located on the outer surface or on the α-helix in the C3 region. In all patients except patient B, Thr283, located in the CD4 binding region (though not directly in contact with it), was inferred to be under positive selection. In patients C and D, distinct sites were under positive selection in this area. Arg476 in patient C, and Thr283 and Asp368 in patient D, were under positive selection and potentially involved in direct receptor binding. A more clearly delimited constraint seems to act on patients E, F and G. In particular, a conformational epitope appeared to be present in patient E and G and formed by Thr278, Asp279 and Ala 281. In patient F, a complex and large area located partially within the CD4 binding site and in a usually highly conserved region immediately next to it was observed to be under positive selection. This region includes Ala281, Trp427, Glu460, Ser461, Glu462 and Leu452 and Leu453. When the IgGb12 heavy chain CDRs structures were superimposed on patient G-derived gp120 3-dimentional visualization, a high number of positively selected sites identified in this patient coincided with residues recognized by this broad neutralizing antibody on the gp120 surface [34].

### Identification of rare mutations

When the amino acid entropy of positively selected sites was studied, the majority of substitutions observed for all patients were between residues present in that same position with a high frequency in the 500 database sequence alignment. Nevertheless, in some patients, rare substitutions seem to have been selected, including E269D, N339H, N339D, N340D, N340K, T341A, N343Q, N343E, A346F, A346Y, T394A, T394I, R476K, R476M. Amino acid frequencies in those positions in the 500 sequence database alignment and how these sites evolved during the observation period are shown in Table 1.

**Table 1:** Evolution of positively selected amino acids that were rarely found in the 500 sequences database.

**AA pos**	**Frequency in the database**	**A**			**B**			**C**			**D**			**E**			**F**			**G**		
		
		**I**	**II**	**III**	**I**	**II**	**III**	**I**	**II**	**III**	**I**	**II**	**III**	**I**	**II**	**III**	**I**	**II**	**III**	**I**	**II**	**III**
***269***	Glu 76.8% **Asp 8.1%**	E			E			E			**D** **15/15**	**D** **15/15**	**D** **10/13**	E			E			**D** **19/19**	**D 9/14**	**D** **9/10**
													E 3/13								E 5/14	E 1/10

***339***	Asn 78.5% **Asp 4.3%** **His 0.5%**	N			N			N 8/13	N 14/15	N 8/14	V			N			K	K	N	N		
								**D** **4/13**	**H** **1/15**	**H** **6/14**												
								**H** **1/13**														

***340***	Lys 32.5% Asn 31.3% **Asp 7.2%**	E			**D** **16/16**	**D** **3/15**	N 10/10	**D**			K			N			N			N		
						N 12/15																

***341***	Thr 91.4% **Ala 5.7%**	T			T			T			T 13/15	T 5/12	T 1/3	T			T			T		
											**A** **2/15**	**A** **7/12**	**A** **12/13**									

***343***	**Asn 2.4%** lys 25.9% Gln 40.5% **Glu 9.6%**	K			K			**N** **2/13**	**N** **14/15**	**N** **5/14**	K 15/15	K 7/12	**E** **3/13**	K 12/12	K 6/8	K 5/14	G 13/13	**E** **14/19**	K 14/14	K 19/19	K 11/11	**E** **2/7**
								K 11/13	K 1/15	K 5/14		**E** **4/12**	K 10/13		R 1/8	Q 8/14		R 5/19				Q 2/10
										R 4/14		T 1/12			**E** **1/8**	R 1/14						

***346***	Val 37.6% Ala 26.8% Ser11.2% **Phe 0.2%** **Tyr 0%**	V			A 5/16	V 15/15	V 10/10	V 4/13	V 15/15	V 10/14	V			V 3/12	V 6/8	V 6/14	V			V 19/19	V 7/11	A 9/9
					V 11/16			A 4/13		A 4/14				**F** **5/12**	**F** **2/8**	**Y** **8/14**					A 4/11	
														A 4/12								

***394***	Thr 81.3% **Iso 3.1%** **Ala 1.7%**	T			T			T 4/13	T 13/15	T 6/14	T			T			T			T		
								**I 9/13**	**I 1/15**	**I 8/14**												
									**A 1/15**													

***476***	Arg 81.3% **Lys 18.4%** **Met 0%**	K			R			R 11/13	R 3/15	R 8/14	R			**M**			R			R		
								**K 2/13**	**K 12/15**	**K 6/14**												

Their frequency in the sequence database and their proportion (number of clones with the mutation/number of clones sequenced) in the viral quasispecies at each time point (I, II, and III) are shown.

### HLA typing

A low- or high-resolution HLA typing was also performed for patient A to E. HLA typing was not possible for patients F and G. Results of HLA typing are shown in Additional file 3.

## Discussion

In the present study, a high-resolution phylogenetic analysis of the gp120 envelope glycoprotein evolution was performed in HIV-1 infected patients with a different pattern of disease progression. All patients under study had never been treated for HIV-1 infection, leaving the host immune system as the only selective force acting on virus evolution and quasispecies selection. Firstly, an analysis was performed to identify putative recombinants. Recombination may occur frequently in vivo in HIV-1 evolution, and artificial chimeric sequences due to PCR crossovers can significantly affect phylogenetic analysis. The PHI test based on the refined incompatibility score was used to overcome this bias with our data set [35]. When recombinant sequences were excluded (about 15%, see materials and methods) from the analysis, the number of sites with a dN/dS value > 1 was reduced in some of the patients. Nevertheless, the number of positively selected sites identified with a Bayesian posterior probability > 0.95 in our datasets was not significantly affected. The best fitting model of evolution was chosen in the phylogenetic reconstruction, and maximum likelihood methods were used to fit codon models of evolution for all patients, to identify positively selected sites, and Bayesian inference was used to estimate virus evolutionary rates. In addition, an HLA typing and a color-grade 3-dimensional visualization of the dN/dS score were used.

Finally, since external branches are subject to substitutions as well as mutational load, which involves random mutations and therefore potentially many nonsynonymous substitutions, we inferred the sites under selection for the internal branches only, using the Fixed Effects Likelihood (FEL) approach [36]. This analysis infers dN and dS for each site and also tests whether dN = dS or not for the sites [36]. All the sites identified with the FEL approach were also identified with the previous methods, further confirming the possibility of identifying sites showing diversifying selection when sequential time points are considered even using cloned sequences. A multiple-step analysis was in fact necessary in the present study to address correctly the evolution of a large portion of the HIV-1 *env *gene, since a high background is expected when the dN/dS score/site is performed in highly variable viral populations under continuous positive selection. In these cases, only sites with high dN/dS ratio and confirmed by Bayesian posterior probability should be taken into consideration [32,37,38]-.

In order to highlight the effect of positive selection on virus evolution, the evolutionary rate was calculated separately in the three codon positions. In the third codon position, mutations are silent in about 70% of all possibly occurring nucleotide changes, and if no selective constraints act on the virus, evolution occurs at a faster rate compared to the first and second codon positions. In all LTNPs, the third codon mutation rate is equal to or lower than that compared to the averaged 1^st ^and 2^nd ^position (p = 0.016), thus being compatible with positive selection [39-41].

The impact of HLA-associated selection pressure on viral evolution has recently been demonstrated at the population level [42-50]. No HLA B57 associated positively selected sites were identified in our patients, but a potential HLA A11 associated epitope was present in patients B, C, and E. Within this epitope, the position 346 exhibited a high dN/dS ratio in all three patients.

Although positive selection was evident in the replicating virus from all subjects, differences were observed between NPs and LTNPs. In subjects A and B (NPs) selective constraints are less intense, in terms of dN/dS score calculated even for the highly selected hotspots (Figure 2 and 3), and are limited to the external surface of the crystal and to the α-helix in the C3 region. These sites and the V3 loop appear to be targets for the immune response in all patients, with a single exception (patient A). This observation is apparently in contrast with the results obtained by other studies, where the C3 alpha helix was observed to be under positive selection for clade C envelopes and only modestly for clade B [27,51]. Although we cannot exclude that differences in the intensity of the immune response against different HIV-1 subtypes exist at these levels, the previous analyses were based on cross-sectional C-clade and B-clade sequence datasets downloaded from HIV-1 databases, thus not reflecting the intra-patient evolutionary dynamics and the heterogeneity of host immune responses during the different phases of HIV-1 infection (or the different patterns of disease progression observed). Other studies analyzed the sequence evolution in infected individuals and showed that the C3 region, including the externally accessible residues, is under strong positive selection both in clade B [24-26] and in HIV-1 subtype C infections [23]. These results may be of particular interest since this antigenic portion of the gp120 molecule has been considered in the development of candidate vaccines [52-56]-.

Many N-linked glycosylation sites were identified to be under positive selection and exposed on the surface in the group of LTNPs and in the 2 NP subjects. In particular N442, R444 and S446, N295, N332, N340, N339 were identified as being potentially involved in the glycan shield that protects the virus against host defences [57]. Interestingly, it has been demonstrated that the neutralizing activity of a human monoclonal antibody, designated as mAb 2G12, is associated with the presence of glycosylation sites at these positions, including 295, 332 and 339 [58-60]. IgG 2G12-like antibodies have previously been detected in LTNP patients by competitive ELISA experiments with high levels in sera associated with the broad neutralizing activity [19]. This observation is in perfect agreement with our data, suggesting that antibodies that bind close to the 2G12 binding site exist in some patients and exert selective pressure on the viral surface.

It has recently been observed that cross-neutralizing activity characterizing a small subset of LTNPs is associated with antibodies recognizing the CD4bs [22]. However, only a few broadly neutralizing human monoclonal antibodies have been isolated at present; among them, only the IgGb12 (directed against the CD-4bs) and mAb 2G12 (recognizing oligomannose residues) target the gp120 [58,61,62]. Notably, 4 out of the 5 LTNP patients exhibit strong selective constraints at the level of the CD4bs. In patient F in particular, an IgGb12 epitope-like area is under strong positive selection (Figure 5). These data document that this epitope can be modified in vivo in response to specific selective pressure. Further analyses are necessary to clarify if mutations in this region may alter the viral RC, thus being able to delay disease progression.

## Conclusion

The present study describes the dynamic evolution of the HIV-1 *env *gene in a subset of LTNP subjects and documents that the CD4bs is under strong selective pressure in the replicating virus of a group of LTNPs and evolves during the course of the disease. These data may be of interest not only for the understanding of the complex HIV-1-host relationships, but also for planning new immune-based strategies against HIV-1 infection.

## Methods

### Patients and sequences

Seven HIV-positive patients, never treated for HIV infection, were selected on the basis of the slope of their CD4+-T-cell counts and the level of HIV-1 viremia (Table 2). Two of them (subjects A and B) were showing a typical progression of HIV infection (TP), with a gradual decline of CD4+ T cells over time (loss of circulating CD4+ T cells per year: subject A, 87; and subject B, 153). The patients designated C, D, E, F, and G were LTNPs with CD4+-T-cell constantly higher than 500 per ml. They were showing the following mean variation/year of circulating CD4+lymphocytes: -31, -24, -2, -10, and +12 respectively). Lengths of infection and sampling dates are shown in Table 2.

**Table 2:** Immunologic and virologic parameters of patients that were selected for the study.

Patient	Timepoint (years from infection)	HIVRNA copies/ml of plasma	CD4+ T cell counts/mm^3^
A	I (9)	28000	561
	
	II(11)	32360	379
	
	III(13)	127200	337

B	I (8.5)	25000	374
	
	II(9.5)	65000	255
	
	III(10.5)	40000	259

C	I(10)	99	1144
	
	II(11)	100	944
	
	III(12)	549	776

D	I(9.5)	2918	993
	
	II(10.5)	3318	735
	
	III(12)	6850	640

E	I(11.5)	1200	795
	
	II(12.5)	2000	720
	
	III(13.2)	3034	527

F	I(8)	2100	645
	
	II(10)	1200	657
	
	III(13)	6393	625

G	I(10)	560	864
	
	II(13)	106	926
	
	III(14)	152	750

Plasma specimens were concentrated by centrifugation at 23,600 × *g *for 1 h at 4°C and RNA was extracted by using a QIAamp viral RNA mini kit (Qiagen, Valencia, CA). The following outer primers were used in the nested PCR amplification reaction: V31 (nucleotides 6939 to 6966 in the pNL4-3 numbering system) and V52 (nucleotides 7803 to 7778). The internal primers were: V32 (nucleotides 7367 to 7340), and V41 (nucleotides 7304 to 7326). The reverse transcription of HIV-1 RNA present in plasma was performed with primer V52 (25 pmol) and 200 U of SuperScript II RNase H-RT (Bethesda Research Laboratories, Gaithersburg, Md.) at 37°C for 60 min in a final volume of 20 μl in the presence of 3.0 mM MgCl_2_, 75 mM KCl, 50 mM Tris (pH 8.3), 10 mM dithiothreitol, 0.5 mM concentrations of each deoxynucleosidetriphosphate (dNTP), and 20 U of recombinant RNasin RNase inhibitor (Promega Corp., Madison, Wis.). An amount of cDNA equivalent to 50–1000 copies of template (as evaluated by HIV-1 RNA copies/ml) was used for PCR amplification. An Expand High Fidelity PCR system (Roche Diagnostic Corporation, Indianapolis, IN) in 1× Expand PCR buffer containing 1.5 mM MgCl_2_, 0.2 mM of each deoxynucleoside triphosphate, and 0.2 μM of V31 and V52 primers was used for the first round. The following cycling conditions were applied: 94°C for 2 min followed by 35 cycles of 94°C for 15 s, 55°C for 30 s, and 68°C for 4 min, with a final extension of 68°C for 10 min. Second-round PCR was performed using 2 μl of the first-round PCR product and primers V32 and V41 under the same conditions used for the first-round PCR.

Only one sample at a time was processed, and clinical samples were amplified in triplicate. Before molecular cloning, a 10-μl aliquot of the amplified product was run on a 10% polyacrylamide gel electrophoresis to screen for the appropriate-sized band (ca. 865 bp); the remaining 90 μl was resolved by electrophoresis on a 1.5% low-melting-point agarose gel (SeaPlaque; FMC BioProducts, Rockland, Maine) in TAE buffer (Tris-acetate, 1 mM EDTA). The DNA fragment was excised from the gel, purified by the QIAquick DNA Clean-Up system (Qiagen GmbH, Hiden, Germany) and cloned into pGEM-T vector (Promega) according to the manufacturer's instructions. After PCR colony screening, 8 to 20 positive clones were selected and the insert was sequenced with primers V31 and V52 on an automatic sequencer (ABI Prism 3100, Appliedbiosystems, Foster City, CA).

For patient A, 15 clones per time point were studied; for patient B 16,15 and 10 clones respectively; for patient C, 13, 15, and 24 clones; for patient D, 15,12 and 13; for patient E, 12, 8, and 14 clones, for patient F, 13, 19, and 14 clones; and for patient G, 19, 15, and 9 clones.

Because some analyses could potentially be affected by recombinats, we tested for recombination using the PHI test implemented in the SplitsTree package version 4.8. Significance of the PHI statistic for the presence of recombination is assessed with the normal approximation of a permutation test where, under the null hypothesis of no recombination, sites along the alignment are randomly permuted to obtain the null distribution of PHI: p < 0.05 indicate significant presence of recombination. About 15% of sequences for each time point/patient were discarded after this analysis.

GenBank accession numbers of sequences are EU329847 – EU330175.

### High resolution phylogenetic analysis & graphical 3-dimensional visualization

Overall, 278 non recombinant genomic-HIV-1 RNA sequences were aligned collectively and individually for each patient, using amino acidic sequences as template for nucleotide alignment by using DAMBE  and manually corrected with BioEdit . A Neighbor-Joining (NJ) tree was reconstructed using the best fitting evolutionary model as evaluated with MODELTEST v3.6 (Posada, D., and K.A. Crandall, 2001) was generated.

To obtain a maximum-likelihood tree topology, a local rearrangement search with the maximum-likelihood method was conducted by starting from the topology of the NJ tree, as implemented in PAUP* . The ratio of transitions to transversions, and the gamma distribution of rate variation among sites were estimated from the data. To evaluate if intra-patient virus evolution showed patterns of positive selection, a ML method was applied by using CODEML implemented in the PAML package  and the substitution rate at individual codon position was also estimated for each patient using the TipDate model as implemented in BEAST . The CODEML program fits various models of codon evolution to sequence data related by a phylogenetic tree, which allow to test for varying selection pressures at individual codon sites. The models of codon evolution differ in their distribution of dN/dS values among codons. Two couples of nested models were employed: M1a vs M2a and M7 vs M8. M1a (neutral/purifying model) allows only two categories of dN/dS across codons and the dN/dS ratio is constrained to be > 0 and < 1 in one category and equal to 1 in the other. Hence, M1a only accommodates neutral evolution. M2a adds an extra class of codons to account for positive selection (i.e., a class of codons with dN/dS > 1). M7 (neutral model) assumes a beta distribution of dN/dS between 0 and 1 with 10 categories to discretize the distribution. M8 adds an extra class of codons with dN/dS > 1 [63]. The likelihoods of the models were than compared using the likelihood ratio test. To allow further definition of HIV-1 env positively selected sites within each patient, all the RNA-sequences amplified and cloned from the same subject, were analysed by using an empirical Bayes approach [64]. The posterior mean dN/dS value per site was calculated and a Bayesian approach was used to identify codons undergoing positive selection with a posterior probability of > 95% or > 99%, using CODEML. To better identify conformational epitopes and sites on the protein surface with possibly distinct roles on disease progression, and distinct patterns of virus evolution driven by host-selective constraints along the C2-V5 region, a graphic colour-grade 3-dimensional visualization the dN/dS score (ratio between non-synonymous/synonymous mutations per site) was generated using PyMol  and the structure of a V3-containing gp120 core [65].

Moreover, to better understand the impact of deleterious mutational load in within-host HIV evolution and its impact on identifying positively selected sites, we performed a ML analysis of varying selection pressures among lineages. In this analysis, we compared model M0 (all branches have the same dN/dS) with an alternative model that allows a different dN/dS for internal and external branches.

Sites under selection along internal branches of reconstructed phylogenetic trees, were inferred using the Fixed Effects Likelihood (FEL) approach implemented in Hy-Phy .

To evaluate the HIV-1 general site-specific inter-exchangeability (site-specific aminoacidic entropy) a collection of 500 aligned env sequences from the Stanford Database was downloaded and analysed with BioEdit accessory applications. When positional homology was not maintained due to the high genetic variability, that site in the alignment was not considered in the analyses.

## Competing interests

The authors declare that they have no competing interests.

## Authors' contributions

FC conceived and coordinated the study and wrote the manuscript; FC and PL did the analyses. MCM, MS, SB and PB carried out the amplification and sequencing. MD did the 3D color-grade mapping on the gp120 structure. GG followed up patients; BM performed the HLA typing; RB, MC discussed the data and reviewed the manuscript. All authors read and approved the final manuscript.

## Supplementary Material

Additional file 1 **Supplementary Table One. Likelihood ratio statistics (2Δl) for comparision of different models of codon evolution.** Click here for file

Additional file 2 **Supplementary Table Two. Analysis of selective pressure among internal and external branches in each patient.** Click here for file

Additional file 3 **Supplementary Table Three. Low- or high-resolution HLA typing.** Click here for file
