# Supplementary material for: Dynamic features of the selective pressure on the human immunodeficiency virus type 1 (HIV-1) gp120 CD4-binding site in a group of long term non progressor (LTNP) subjects
Source: Retrovirology. 2009 Jan 15;6:4. doi: 10.1186/1742-4690-6-4 (PMC2639529; doi:10.1186/1742-4690-6-4)
Supplement: Additional file 1 — Supplementary Table One. Likelihood ratio statistics (2Δl) for comparision of different models of codon evolution. [file 1742-4690-6-4-S1.doc]

**Supplementary table 1.** Likelihood ratio statistics (2Δl) for comparision of different models of codon evolution. Df=degrees of freedom.

|  | Log likelihood  Model 1A | Log likelihood  Model 2A | 2*ΔLog *L*:  M1A vs. M2A | Significance  (df:2) | Log likelihood  Model M7 | Log likelihood  Model M8 | 2*ΔLog *L*:  M1A vs. M2A | Significance  (df:2) |
| --- | --- | --- | --- | --- | --- | --- | --- | --- |
| ***A*** | -2781.82 | -2762.82 | 38,00 | <0.001 | -3431.23 | -3394.52 | 73.42 | <0.001 |
| ***B*** | -2918.75 | -2910.28 | 16,94 | <0.001 | -3135.90 | -3120.13 | 31.54 | <0.001 |
| ***C*** | -2725.71 | -2707.66 | 36,10 | <0.001 | -2878.02 | -2854.00 | 48.04 | <0.001 |
| ***D*** | -2504.72 | -2456.14 | 97,16 | <0.001 | -2941.40 | -2880.56 | 121.68 | <0.001 |
| ***E*** | -3007.09 | -2978.51 | 57,16 | <0.001 | -3221.58 | -3182.10 | 78.96 | <0.001 |
| ***F*** | -2375.46 | -2356.68 | 37,56 | <0.001 | -2576.22 | -2540.92 | 70.60 | <0.001 |
| ***G*** | -2343.75 | -2315.02 | 57,46 | <0.001 | -2824.96 | -2797.79 | 54.34 | <0.001 |
