# Supplementary material for: Dynamic features of the selective pressure on the human immunodeficiency virus type 1 (HIV-1) gp120 CD4-binding site in a group of long term non progressor (LTNP) subjects
Source: Retrovirology. 2009 Jan 15;6:4. doi: 10.1186/1742-4690-6-4 (PMC2639529; doi:10.1186/1742-4690-6-4)
Supplement: Additional file 2 — Supplementary Table Two. Analysis of selective pressure among internal and external branches in each patient. [file 1742-4690-6-4-S2.doc]

**Supplementary table 2.**

Analysis of selective pressure among internal and external branches in each patient. dN/dS estimates were almost always higher on external branches compared to internal branches. For all patients, we also tested a model that allowed for different dN/dS on external branches, internal branches within patients and internal branches representing evolution between patients. This did not fit the data significantly better (2*ΔLog L = 0.39).

| Patient | dN/dS (M0) | dN/dS (int – ext) | 2*ΔLog *L* |
| --- | --- | --- | --- |
| A | 0.35 | 0.48 - 0.28 | 4.72* |
| B | 0.44 | 0.56 - 0.38 | 2.27 |
| C | 0.68 | 0.73 - 0.64 | 0.16 |
| D | 0.99 | 1.27 - 0.87 | 1.10 |
| E | 0.47 | 0.86 - 0.32 | 13.83** |
| F | 0.80 | 0.52 - 1.01 | 3.65* |
| G | 0.56 | 0.54 - 0.58 | 0.04 |
| All | 0.59 | 0.46 - 0.78 | 27.68** |
