# Supplementary material for: Dynamic features of the selective pressure on the human immunodeficiency virus type 1 (HIV-1) gp120 CD4-binding site in a group of long term non progressor (LTNP) subjects
Source: Retrovirology. 2009 Jan 15;6:4. doi: 10.1186/1742-4690-6-4 (PMC2639529; doi:10.1186/1742-4690-6-4)
Supplement: Additional file 3 — Supplementary Table Three. Low- or high-resolution HLA typing. [file 1742-4690-6-4-S3.doc]

**Supplementary Table 3.**

Low- or high-resolution HLA typing. N.D.=analysis not performed.

| Patient | Molecular HLA typing |
| --- | --- |
| A | HLA-A*24, HLA-A*26, HLA-B*13, HLA-B*18, HLA-Cw*06, HLA-Cw*07, HLA-DRB1*11, HLA-DRB1*13, HLA-DRB3*01, HLA-DRB3*02, HLA-DQB1*03, HLA-DQB1*06 |
| B | HLA-A*11, HLA-A*24, HLA-B*35, HLA-B*55, HLA-Cw*01, HLA-Cw*04, HLA-DRB1*01, HLA-DRB1*15, HLA-DQB1*0501, HLA-DQB1*06 |
| C | HLA-A*11, HLA-A*29, HLA-B*35, HLA-B*39, HLA-Cw*04, HLA-Cw*12, HLA-DRB1*08, HLA-DRB1*1001, HLA-DQB1*0402, HLA-DQB1*0501 |
| D | HLA-A*29, HLA-A*33, HLA-B*1402, HLA-B*35, HLA-Cw*04, HLA-Cw*08, HLA-DRB1*01, HLA-DRB1*13, HLA-DRB3*01, HLA-DQB1*03, HLA-DQB1*0501 |
| E | HLA-A*11, HLA-A*33, HLA-B*1402, HLA-B*35, HLA-Cw*04, HLA-Cw*08, HLA-DRB1*01, HLA-DRB1*11, HLA-DRB3*02, HLA-DQB1*03, HLA-DQB1*0501 |
| F | N.D. |
| G | N.D. |
